# Sex‐allocation conflict and sexual selection throughout the lifespan of eusocial colonies

**DOI:** 10.1111/evo.13746

**Published:** 2019-05-03

**Authors:** Piret Avila, Lutz Fromhage, Laurent Lehmann

**Affiliations:** ^1^ Department of Ecology and Evolution University of Lausanne Biophore 1015 Lausanne Switzerland; ^2^ Department of Biological and Environmental Science University of Jyväskylä P.O. Box 35 Jyväskylä FI‐40014 Finland

**Keywords:** Conflict, life‐history strategy, optimal resource allocation, social insects, sex allocation

## Abstract

Models of sex‐allocation conflict are central to evolutionary biology but have mostly assumed static decisions, where resource allocation strategies are constant over colony lifespan. Here, we develop a model to study how the evolution of dynamic resource allocation strategies is affected by the queen‐worker conflict in annual eusocial insects. We demonstrate that the time of dispersal of sexuals affects the sex‐allocation ratio through sexual selection on males. Furthermore, our model provides three predictions that depart from established results of classic static allocation models. First, we find that the queen wins the sex‐allocation conflict, while the workers determine the maximum colony size and colony productivity. Second, male‐biased sex allocation and protandry evolve if sexuals disperse directly after eclosion. Third, when workers are more related to new queens, then the proportional investment into queens is expected to be lower, which results from the interacting effect of sexual selection (selecting for protandry) and sex‐allocation conflict (selecting for earlier switch to producing sexuals). Overall, we find that colony ontogeny crucially affects the outcome of sex‐allocation conflict because of the evolution of distinct colony growth phases, which decouples how queens and workers affect allocation decisions and can result in asymmetric control.

Eusocial Hymenopteran colonies may superficially appear to function as single organisms, where queens and workers could be viewed as the germinal and somatic tissues of multicellular organisms (Macevicz and Oster 1976). However, such individuals are usually not clonal, whereby some genes, for instance those influencing sex allocation or reproductive ability of workers, can experience diverging selection pressures in different individuals (e.g., Hamilton 1967; Bourke and Franks 1995; Haig 2003; Ratnieks et al. 2006).

One of the most intensively studied genetic conflicts is the queen‐worker conflict over sex allocation. In an outbred haplodiploid population where each colony is headed by a singly mated queen, natural selection on resource allocation strategies favors alleles in queens that code for equal resource allocation to males and (sexual) females and alleles in workers that code for a 3:1 (sexual females to males) allocation ratio (e.g., Trivers and Hare 1976; Frank 1998; West 2009). Factors such as multiple related queens per colony and multiple matings by the queen reduce the extent of the genetic conflict over sex allocation because they reduce relatedness asymmetries between individuals within colonies (e.g., Frank 1998; Ratnieks et al. 2006; West 2009).

The long‐term evolutionary “outcome” of the sex‐allocation conflict—the uninvadable resource allocation schedule, is determined by the mechanisms through which the opposing “parties” can influence how colony resources are allocated into producing individuals of different types. In a colony founded by a single queen, there are two opposing parties: the genes in the workers and the genes in the colony‐founding queen. The genetic control over resource allocation decisions can be achieved through different genetic, behavioral, and physiological processes (Beekman and Ratnieks 2003; Mehdiabadi et al. 2003; Helanterä and Ratnieks 2009). Hereinafter, if one party fully determines a given resource allocation trait, then this party is said to be “in control” of that trait (here, “in control” has a related but more restricted meaning than “having power” as in, e.g., Beekman and Ratnieks 2003). In general, there are reasons to expect that the genes in the queen and workers simultaneously control different resource allocation decisions because both parties are known to have means to control different resource allocation decisions and selection for a party to seize control over a resource allocation decision can be strong if there are means to do so (Trivers and Hare 1976; Bourke and Franks 1995; Helanterä and Ratnieks 2009). Furthermore, it is often considered most likely that the genes in the queen determine the primary sex‐allocation ratio (allocation of resources to females vs. males) and the workers control the developmental fate of the female eggs (Trivers and Hare 1976; Bourke and Franks 1995; Helanterä and Ratnieks 2009). Hereinafter, we refer to this scenario as “mixed control.”

Theoretical models of sex‐allocation conflict provide three important insights into fundamental questions in evolutionary biology (e.g., Pamilo 1991a; Bourke and Chan 1999; Bourke and Ratnieks 1999; Reuter and Keller 2001; Reuter et al. 2004; Pen and Taylor 2005). First, they provide clear predictions that allow to test how relatedness affects selection on social traits (Crozier and Pamilo 1996). Second, they allow to predict which party is in control of the underlying resource allocation decisions, given that one has sex‐allocation data. Third, they enable to predict to what extent the conflicts can be “resolved” (sensu Ratnieks et al. 2006, i.e., conflict outcome with modest colony‐level costs) under various assumptions about the mechanisms of genetic control over the resource allocation decisions. However, all of the aforementioned models consider static allocation decisions without explicitly taking colony ontogeny into account. Nevertheless, it is known that many annual eusocial insect species (e.g., vespid wasps, bumble bees, and sweat bees) grow in two distinct phases (see references in Mitesser et al. 2007a; Crone and Williams 2016). That is, in the beginning of the season only workers are produced (ergonomic phase) followed by a drastic shift into exclusive production of males and future queens (reproductive phase). This life‐history schedule was shown to be an evolutionary outcome in annual eusocial colonies assuming clonal reproduction by Macevicz and Oster (1976). However, only a few theoretical studies (Bulmer 1981; Ohtsuki and Tsuji 2009) have considered sexually reproducing species (thereby including the possibility of genetic conflicts) and time‐dependent resource allocation decisions in the context of colony life‐history. The importance of colony ontogeny in studying within‐colony conflict was demonstrated by Ohtsuki and Tsuji (2009) who showed (in the context of worker policing) that the expression of conflict depends on the phase of colony ontogeny.

In his seminal work, Bulmer (1981) showed using a dynamic allocation model (i.e., time‐dependent decisions) that the sex‐allocation conflict can have a detrimental effect on colony productivity (sexual biomass) under mixed control because relatively few resources are allocated into producing workers. Indeed, he predicted that the production of workers is expected to halt earlier under mixed control, but he did not consider the entire colony ontogeny and his predictions relied on some additional restrictive assumptions. For example, he assumed that the worker generations do not overlap within a season (i.e., a colony grows in separate generations of workers within a season) and that sexuals can only mate at the very end of the season. Hence, theoretical understanding of the life‐history decisions of eusocial colonies has mostly relied on the assumption of clonal reproduction with no genetic conflicts (Macevicz and Oster 1976; Mitesser et al. 2007a).

The importance of considering dynamic resource allocation decisions for studying within‐colony conflict is demonstrated by the fact that the static and dynamic resource allocation models can make contradicting predictions about which party wins the sex‐allocation conflict under mixed control (Bulmer 1981; Reuter and Keller 2001). Indeed, the static resource allocation model by Reuter and Keller (2001) predicts a sex‐allocation ratio under mixed control that is intermediate between the evolutionary predictions corresponding to worker and queen control. In contrast, Bulmer's (1981) dynamic model predicts that the queen wins the sex‐allocation conflict by laying only haploid eggs at the penultimate generations causing the colony to die one generation before the end of the season if the sex‐allocation ratio in the population is female‐biased. However, the generality of Bulmer's predictions is limited due to the aforementioned restrictive assumptions of his model.

Furthermore, in another study assuming queen control of resource allocation traits and the possibility of sexuals to mate before the end of the season, Bulmer (1983) showed that sexual selection on males will lead to protandry (males being produced before sexual females) if mating can occur over some period of time. Indeed, sexual selection may thus play an important role for colony ontogeny because protandry is found among many annual eusocial insects, for example, in paper wasps and bumble bees (Strassmann and Hughes 1986; Bourke 1997). Evolution of protandry however contradicts the earlier model by Bulmer (1981) for mixed control because it predicted that males are produced in the very end of the season. Hence, there are no theoretical predictions for time‐dependent colony resource allocation decisions and conflicts under mixed control, where individuals can mate over a finite period of time during the season with sexual selection occurring throughout.

In this article, we address the limitations of previous studies by developing a dynamic resource allocation model where we consider three alternative scenarios of genetic control of resource allocation decisions: queen control, worker control, and mixed control; and two alternative scenarios of dispersal of sexuals: delayed dispersal (all sexuals simultaneously disperse at the end of the season to mate) and direct dispersal (sexuals disperse immediately after eclosion to mate). In light of previous work, the purpose of this article is to address the following questions: (i) How does conflict affect colony growth? (ii) How does sexual selection affect the order at which sexuals are produced? (iii) Which party wins the sex‐allocation conflict for different scenarios of dispersal of sexuals?

## Model

### LIFE CYCLE

We consider a seasonal population of haplodiploid eusocial insects consisting of a large (ideally infinite) number of colonies or breeding sites each occupied by a mated queen. The life cycle over a season is assumed to consist of the following four events. (1) *Reproduction*: at the start of the season of total length *T*, each queen occupying one of the *n* breeding sites initiates a colony that can grow throughout the season, and where workers, males, and future queens can be produced. (2) *Dispersal*: sexuals disperse out of their natal colony, such that no inbreeding, local mate competition, or local resource competition takes place; we consider two alternative scenarios for the timing of dispersal (to be detailed below). (3) *Mating*: random mating occurs and all queens mate exactly with M≥1 males. (4) *Regulation*: all individuals die at the end of the season, except (juvenile) queens who randomly compete for the *n* breeding slots to initiate colonies of the next generation.

### DISPERSAL AND MATING

The two dispersal scenarios are as follows: (i) delayed dispersal, where sexuals all disperse at the same time at the end of the season, and (ii) direct dispersal, where sexuals disperse immediately after being produced. Females mate immediately with *M* males in the mating pool after which they will exit the mating pool. In contrast, males continue on mating until they die. Hence, the mating success of a male depends on his mortality rate and the availability of mating females. To gain fitness, females have to survive until the end of the season, whereas males have to inseminate females who survive until the end of the season.

### COLONY GROWTH AND PRODUCTION OF SEXUALS

We model explicitly colony population dynamics during stage (1) of the life cycle. To describe our model, we start by considering that the population is monomorphic for all phenotypes, and we will later introduce variation and selection. The size of a focal colony at time t∈[0,T] in the (monomorphic) population is $y_{w}\left(t\right)$, which gives the number of sterile workers (including the colony‐founding queen, who has been counted as a worker) in the colony at time *t*. In addition, by time *t*, the colony has produced $y_{q}\left(t\right)$ surviving (juvenile) queens and $y_{m}\left(t\right)$ surviving (juvenile) males. By the term “juvenile” we only want to emphasize that these sexual individuals are regarded as offspring in the current generation and that they will reproduce in the next generation. For simplicity, we assume that all individuals are equally costly to produce, which allows to equate the investment allocation ratio to the numerical sex ratio. However, the assumption of equal production cost has no fundamental effect on the evolutionary process because selection acts only on total investment in the sexes and not on their numbers and hence is independent of the production costs of different individuals (West 2009).

Workers acquire resources from the environment to produce offspring. Let *b* denote the individual productivity rate of a worker (i.e., the net rate at which a worker acquires resources for the colony, measured in individuals produced per unit time). For simplicity, we assume that the availability of resources in the environment is constant over time and the rate at which resources are acquired scales linearly with the colony size (i.e., *b* is constant). The latter assumption implies that there are enough resources in the environment to sustain constant per worker rate of resource acquisition and the egg‐laying rate of the queen is constrained only by the resources available to the colony.

The number $y_{k}\left(t\right)$ of type k∈{w,q,m} individuals alive at time *t* that were produced in the focal colony is assumed to change according to

(1) $$\frac{\;dy_{k}\left(t\right)}{\;dt}=ba_{k}\left(t\right)y_{w}\left(t\right)-\mu_{k}y_{k}\left(t\right),\;y_{k}\left(0\right)=y_{k,0},$$

where $a_{k}\left(t\right)$ is the fraction of resources allocated into producing type *k* individuals at time *t*, $\mu_{k}$ is the mortality rate of individuals of type *k*, and $y_{k,0}$ is the number of type *k* individuals in the colony in the beginning of the season. The initial condition (number of individuals at the beginning of the season) for the colony is $y_{w,0}=1$ (the colony‐founding queen is counted as a worker because she can, for example, recover some resources from her body fat), $y_{q,0}=0$ (no juvenile queens), and $y_{m,0}=0$ (no juvenile males). Note that the number of juvenile queens $y_{q}\left(t\right)$ and males $y_{m}\left(t\right)$ are counted regardless if they have dispersed from the colony.

It will turn out to be useful to keep track of the number of queens that the males from a focal colony have inseminated. Let y iq (t) be the expected number of females alive at time *t*, who have been inseminated by males from a focal colony, given that females mate only once (i.e., under a monandrous mating system, M=1) and it changes according to

(2) dy iq (t)dt=0,fort<T,withy iq (T)=ym(T)yq(T)ym(T)(delayeddispersal),ym(t)baq(t)yw(t)ym(t)−μqy iq (t),y iq (0)=0(directdispersal).

Under delayed dispersal, all females are inseminated at time t=T, where a total number of $ny_{m}\left(T\right)$ males compete for $ny_{q}\left(T\right)$ females. Hence, the mating success of a male produced in a focal colony is $y_{q}\left(T\right)/y_{m}\left(T\right)$, and the number of males in that colony at the end of the season is $y_{m}\left(T\right)$. Under direct dispersal, females mate immediately after being produced, whereby at time *t* a total number of $nba_{q}\left(t\right)y_{w}\left(t\right)$ females are available to mate (after which they will leave the mating pool). In contrast, males stay in the mating pool, hence at time *t*, an average number of $ny_{m}\left(t\right)$ males compete for the access to females. Therefore, the mating rate of a male produced in a focal colony is $ba_{q}\left(t\right)y_{q}\left(t\right)/y_{m}\left(t\right)$ at time *t* and the last term in the second line of equation (2) takes into account the mortality of the inseminated females. If females mate *M* times, then there are on average *M* times more matings available to males at any given time. Hence, the number of (surviving) females at time *t*, who have been inseminated by males from a focal colony is My iq (t) in a population where females mate *M* times.

### RESOURCE ALLOCATION TRAITS

We assume that the allocation schedule, $a_{k}\left(t\right)$ (k∈{w,q,m}), that governs the dynamics of individuals produced in the focal colony (recall equation 1), is controlled by two traits:

(3) $$\begin{array}{ccc} a_{w}\left(t\right) & & =v_{f}\left(t\right)\left(1-v_{q}\left(t\right)\right),\;a_{q}\left(t\right)=v_{f}\left(t\right)v_{q}\left(t\right),\\ & & a_{m}\left(t\right)=\left(1-v_{f}\left(t\right)\right). \end{array}$$

The first trait $0\leq v_{f}\left(t\right)\leq 1$ is the proportion of resources allocated to producing females (individuals destined to become workers or queens) at time *t*. The second trait $0\leq v_{q}\left(t\right)\leq 1$, gives the proportion of resources allocated to producing queens from resources allocated to females at time t∈[0,T]. Thus, $\left(1-v_{f}\left(t\right)\right)$ is the proportional allocation to males and $\left(1-v_{q}\left(t\right)\right)$ is the proportional allocation of resources directed to producing workers from resources allocated to females.

Our aim is to investigate the evolution of the resource allocation schedule during the whole colony ontogeny, that is, the evolution of $v={\left\{v_{f}\left(t\right),v_{q}\left(t\right)\right\}}_{t\in [0,T]}$. In species where workers are sterile (as assumed here) the queen is often thought to control the allocation between females and males (trait *v*_f_) because she decides at which rate she lays female and male eggs. However, the genes in the workers can influence *v*_f_, if they are able to redirect resources from male brood to female brood (Sundström et al. 1996; Chapuisat et al. 1997), but for simplicity we do not consider this scenario in our article. In many species, the genes in the workers control the developmental fate of the female larvae (trait *v*_q_) by differential feeding, as the diet provided to the larvae by workers determines the caste of the female offspring (Ratnieks et al. 2006; Schwander et al. 2010; Berens et al. 2015). However, in some species, queens can also alter the caste determination of females by producing different types of diploid eggs (Wheeler 1986). It is believed that in many eusocial insects, the queen and the workers are in control of different resource allocation decisions simultaneously and it is often considered most likely that the queen determines the primary sex ratio (ratio of female to male eggs), whereas the workers control the developmental fate of the female eggs (Trivers and Hare 1976; Bourke and Franks 1995; Helanterä and Ratnieks 2009). Hence, in light of the empirical evidence of genetic control of resource allocation decisions, we will examine three possible scenarios of genetic control over these traits: queen control (i.e., the genes in the queen determine resource allocation decisions), worker control (i.e., the genes in the queen determine resource allocation decisions), and mixed control, where the genes in the queen control *v*_f_ (the proportional investment into females vs. males) and the genes in the workers control *v*_q_ (the proportional investment into new queens versus workers). Our assumptions of the genetic control are in accordance with the corresponding assumptions of the static resource allocation model by Reuter and Keller (2001), where they also considered these three scenarios with the corresponding static traits.

To analyze the long‐term evolution of the resource allocation traits, we perform an evolutionary invasion analysis (see Supporting Information Section 1 for more information). That is, we consider the fate (invasion or extinction) of a single mutant allele (an allele determines the entire allocation schedule, that is, a trajectory of the trait over t∈[0,T]) introduced into a population of resident individuals and ask what is the (candidate) uninvadable allocation schedule $v^{*}={\left\{v_{f}^{*}\left(t\right),v_{q}^{*}\left(t\right)\right\}}_{t\in [0,T]}$; namely, the allocation schedule resistant to invasion by any mutant schedule that deviates from $v^{*}$. We determine the (candidate) uninvadable allocation schedule $v^{*}$ analytically using Pontryagin's maximum principle (see Supporting Information Sections 3– 6), which gives a necessary condition for optimality, and we confirm these results numerically using GPOPS‐II (Patterson and Rao 2014), which gives support to the attainability of the uninvadable schedules (see Supporting Information Section 11).

## Results

### MARGINAL VALUE, RELATEDNESS ASYMMETRY, AND POTENTIAL FOR CONFLICT

#### Dynamic marginal value result

Consider a mutant allocation schedule $u={\left\{u_{f}\left(t\right),u_{q}\left(t\right)\right\}}_{t\in [0,T]}$ that deviates slightly from a candidate uninvadable schedule $v^{*}$, such that a trait $u_{\tau }\left(t\right)$ (τ∈{f,q}) can be expressed as

(4) $$u_{\tau }\left(t\right)=v_{\tau }^{*}\left(t\right)+\varepsilon_{\tau }\eta_{\tau }\left(t\right),$$

where $\eta_{\tau }\left(t\right)$ is a feasible phenotypic deviation from the resident trait $v_{\tau }^{*}\left(t\right)$ and $\varepsilon_{\tau }\ll 1$ scales the magnitude of this variation. By a feasible phenotypic deviation we mean any deviation $\eta_{\tau }\left(t\right)$ such that the mutant strategy $u_{\tau }\left(t\right)$ satisfies the constraints of the model (i.e., $0\leq u_{\tau }\left(t\right)\leq 1$ for all t∈[0,T], e.g., see Sydsæter et al. 2008, p. 129 and 308).

Let us now denote by $y_{k}\left(u\right)\equiv y_{k}\left(T\right)$ the number of type k∈{q, iq } individuals at the end of the season, where the resident allocation schedule v in equations (1) and (2) has been replaced by the mutant allocation schedule u. Then, the first‐order condition for a schedule $v^{*}$ to be uninvadable when party c∈{q,w} is in control of the trait of type τ∈{f,q} can be written as

(5) dy iq (u)dετ|εf=εq=0+Rcdyq(u)dετ|εf=εq=0≤0,

which has to hold for all feasible phenotypic deviations $\eta_{\tau }$. For mixed control, the inequality (5) must hold simultaneously for each trait being under the control of the respective party (see Supporting Information Sections 2.1– 2.2 for a proof). Here, $\;dy_{k}\left(u\right)/\;d\varepsilon_{\tau }$ is a Gâteaux derivative (a type of functional derivative, e.g., Troutman 2012, pp. 45–50, Luenberger 1997, pp. 171–178, see also Supporting Information Section 2.1) measuring the change in the number of individuals $y_{k}\left(u\right)$ of type k∈{q, iq } produced by the end of the season in a mutant colony (and we here emphasized that this number depends on the whole allocation schedule, recall equations 1 and 2) due to the infinitesimal deviation $\varepsilon_{\tau }\eta_{\tau }\left(t\right)$ of the trait of type τ throughout the entire season t∈[0,T]. Equation (5) is not a strict equality because the (pointwise) selection gradient does not vanish when a population evolves towards the boundary of the set of possible allocation strategies (e.g., when only workers are produced over some time span, see Supporting Information Sections 2 and 3 for more details, especially Sections 2.3 and 2.4 for pointwise selection gradient and first‐order condition).

The first‐order condition (5) says that at the uninvadable state, the marginal (gene) fitness return (“marginal return” for short) from allocating more resources to male production (measured in the currency of inseminated queens) cannot exceed the marginal loss from allocating less resources to queen production weighted by $R_{c}$, which is the so‐called *relatedness asymmetry* (Boomsma and Grafen 1991, p. 386) defined as

(6) $$R_{c}=\frac{\alpha_{q}^{\circ }r_{q,c}^{\circ }}{\alpha_{m}^{\circ }r_{m,c}^{\circ }},$$

where $\alpha_{s}^{\circ }$ is the (neutral) reproductive value of all individuals of class s∈{q,m}, that is, the probability that a gene taken in the distant future descends from an individual in class s∈{q,m} and $r_{s,c}^{\circ }$ is the (neutral) coefficient of relatedness between an individual of type s∈{q,m} and an average individual whose genes are in control of the resource allocation trait. In Supporting Information Section 2 (equations S30–S32), we detail that the relatedness asymmetry can be interpreted as giving the ratio of sex‐specific (queen/male) contributions, of genes in party *c*, to the gene pool in the distant future (under a neutral process). For haplodiploids the relatedness asymmetry is $R_{q}=1$ (queen control) and $R_{w}=\left(2+M\right)/M$ (worker control).

Equation (5) is a generalized formulation of Fisher's (1930) theory of equal allocation (under queen control) and the standard static marginal value result of sex‐allocation theory (e.g., Taylor and Frank 1996, equation 22). The novelty of equation (5) is that it results from a dynamic model, where the marginal return of producing an individual is time‐dependent, and natural selection favors an allocation schedule that produces males and queens in such a way that the ratio of surviving inseminated queens and produced queens is equal to the relatedness asymmetry. Note that equation (5) does not directly give the ratio of total amount of resources invested (“overall investment” ratio) in each sex, which depends on the characteristics of the life cycle. Furthermore, we show that the overall investment ratios can depart from classic static results of sex‐allocation theory under direct dispersal in our model.

#### Proportional relatedness asymmetry

It follows from the first‐order condition that the marginal value result is given by the relatedness asymmetry, that is, the ratio of sex‐specific asymptotic contributions to the gene pool (equation 5). However, it will turn out to be useful to define the proportional contribution of genes of party *c* through queens to the gene pool in the distant future, that is,

(7) $$P_{c}=\frac{R_{c}}{1+R_{c}},$$

which can be thought of as a proportional relatedness asymmetry. This quantity evaluates to $P_{q}=1/2$ (queen control) and $P_{w}=\left(2+M\right)/\left(2\left(1+M\right)\right)$ (worker control), and it is equal to the (overall) uninvadable proportional allocation into females according to the classical static models of sex‐allocation theory under single‐party control (Trivers and Hare 1976; Boomsma and Grafen 1991; Reuter and Keller 2001).

The conflict between workers and the queen is absent when the proportional relatedness asymmetries for queens and males are equal, that is, $P_{w}/P_{q}=1$. However, when $P_{w}/P_{q}>1$, then future queens are more valuable to workers than to the queen in contributing genes to the gene pool in the distant future. Hence, the ratio

(8) $$C=\frac{P_{w}}{P_{q}}$$

can be interpreted as the potential for conflict. In other words, whenever C≠1, then there is potential for conflict between the queen and the workers over sex allocation. In haplodiploids, the potential for conflict C=C(M)=(2+M)/(1+M) decreases with the increase in polyandry *M* (Ratnieks and Boomsma 1995) because $P_{w}\to P_{q}$ with the increase in queen mating frequency. Hence, the potential conflict C(1)=1.5 is maximal when the queen mates once. It turns out that the proportional relatedness asymmetry $P_{c}$ and the potential for conflict *C* are key quantities describing the properties of the uninvadable allocation schedule $u^{*}$, to which we next turn.

### THE CANDIDATE UNINVADABLE RESOURCE ALLOCATION SCHEDULE

To determine how selection shapes the colony growth schedule, we need to determine the uninvadable allocation schedule $v^{*}$ that satisfies the first‐order condition (recall equation 5). We now present this schedule assuming equal mortality in (juvenile) queens and males (i.e., $\mu_{q}=\mu_{m}=\mu_{r}$) and later discuss the relaxation of this assumption.

#### The colony growth schedule

The uninvadable allocation schedule $v^{*}$ consists of two phases: (i) *the ergonomic phase* ($t\in [0,t_{c,1}^{*}]$) during which workers are produced and (ii) *the reproductive phase* ($t\in [t_{c,1}^{*},T]$) during which sexual offspring are produced (see Supporting Information Sections 5 and 6 for derivations). Here, $t_{c,1}^{*}$ marks the switching time from the ergonomic phase to the reproductive phase and the subscript c∈{w,q, mx } emphasizes the scenario of genetic control. Resource allocation during the reproductive phase depends on the scenario of dispersal of sexuals: (i) under delayed dispersal, resources should be allocated such that the sex‐allocation ratio at the end of the season is given by the relatedness asymmetry $R_{c}$ and (ii) under direct dispersal, males are produced before queens. The switching time $t_{c,2}^{*}\in \left(t_{c,1}^{*},T\right)$ from male production to queen production depends on the scenario of genetic control c∈{w,q, mx } and the sex‐allocation ratio is more male biased than under delayed dispersal.

**Figure 1:**
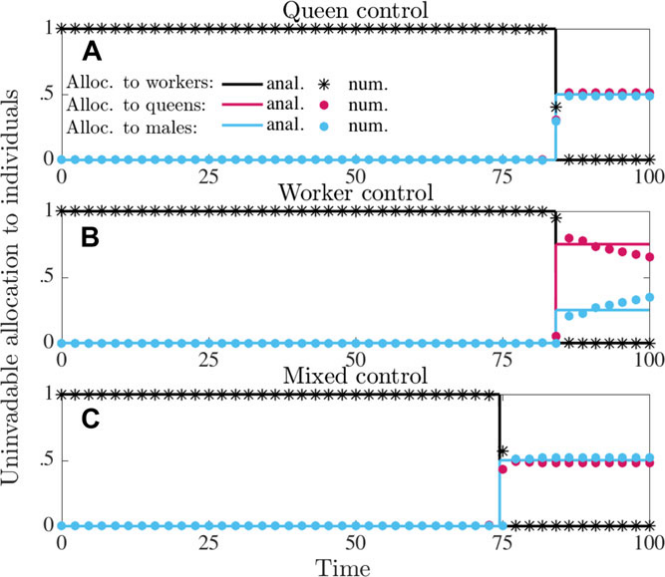
Uninvadable proportional allocation (under delayed dispersal) to workers $a_{w}^{*}\left(t\right)=v_{f}^{*}\left(t\right)\left(1-v_{q}^{*}\left(t\right)\right)$ (black), queens $a_{q}^{*}\left(t\right)=v_{f}^{*}\left(t\right)v_{q}^{*}\left(t\right)$ (red), and males $a_{m}^{*}\left(t\right)=\left(1-v_{f}^{*}\left(t\right)\right)$ (blue). Solid lines are analytically predicted results and the correspondingly colored symbols represent the numerical results. Panel (a) queen control; panel (b) worker control; panel (c) mixed control. Proportional allocation to queens and males exactly match for queen and mixed control, which is why red lines do not appear in the corresponding panels. Note that the numerical results slightly deviate from the analytical results because any strategy that gives the sex ratio (queens to males) at the end of the season, equal to relatedness asymmetry $R_{c}$ of the party in control of $v_{f}\left(t\right)$ has equal invasion fitness (see Fig. 3). Parameter values: M=1, that is, C=1.5 (queen monandry), b=0.07, $\mu_{w}=0.015$, $\mu_{q}=\mu_{m}=0.001$, T=100.

**Figure 2:**
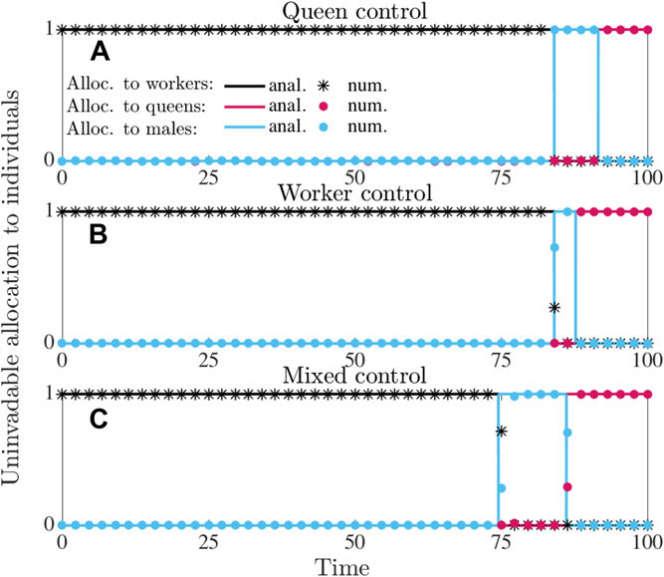
Uninvadable proportional allocation (under direct dispersal) to workers $a_{w}^{*}\left(t\right)=v_{f}^{*}\left(t\right)\left(1-v_{q}^{*}\left(t\right)\right)$ (black), queens $a_{q}^{*}\left(t\right)=v_{f}^{*}\left(t\right)v_{q}^{*}\left(t\right)$ (red), and males $a_{m}^{*}\left(t\right)=\left(1-v_{f}^{*}\left(t\right)\right)$ (blue). Solid lines are analytically predicted results and the correspondingly colored symbols represent the numerical results. Panel (a) queen control; panel (b) worker control; panel (c) mixed control. Parameter values: M=1, that is, C=1.5 (queen monandry), b=0.07, $\mu_{w}=0.015$, $\mu_{q}=\mu_{m}=0.001$, T=100.

**Figure 3:**
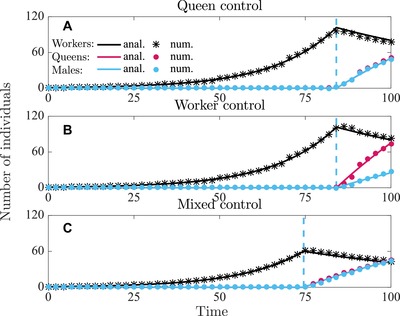
Number of individuals produced in a colony following the uninvadable resource allocation schedule $v^{*}$ under delayed dispersal. Number of workers (black), queens (red), males (blue). Solid lines are analytically predicted results and the correspondingly colored symbols represent the numerical results. Panel (a) queen control; panel (b) worker control; panel (c) mixed control. Parameter values: M=1, that is, C=1.5 (queen monandry), b=0.07, $\mu_{w}=0.015$, $\mu_{q}=\mu_{m}=0.001$, T=100.

**Figure 4:**
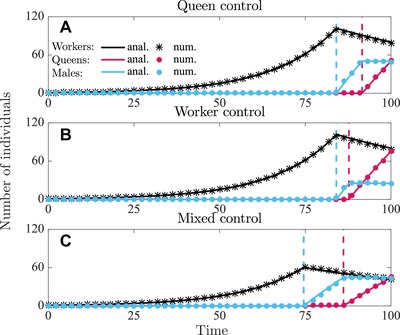
Number of individuals produced in a colony following the uninvadable resource allocation schedule $v^{*}$ under direct dispersal. Number of workers (black), queens (red), males (blue). Solid lines are analytically predicted results and the correspondingly colored symbols represent the numerical results. Panel (a) queen control; panel (b) worker control; panel (c) mixed control. Parameter values: M=1, that is, C=1.5 (queen monandry), b=0.07, $\mu_{w}=0.015$, $\mu_{q}=\mu_{m}=0.001$, T=100.

In Figures 1 and 2, we have depicted the analytically and numerically determined uninvadable allocation schedules $u^{*}$ in terms of proportional allocation to workers $a_{w}^{*}\left(t\right)=v_{f}^{*}\left(t\right)\left(1-v_{q}^{*}\left(t\right)\right)$, queens $a_{q}^{*}\left(t\right)=v_{f}^{*}\left(t\right)v_{q}^{*}\left(t\right)$, and males $a_{m}^{*}\left(t\right)=\left(1-v_{f}^{*}\left(t\right)\right)$ and in Figures 3 and 4 we have depicted the respective number of (surviving) individuals (assuming queen monandry (M=1)).

#### Production of workers in the ergonomic phase

The switching time $t_{c,1}^{*}$ from the ergonomic to the reproductive phase determines the overall amount of resources allocated to workers versus sexuals and it depends on the scenario of genetic control over the resource allocation traits, namely,

(9) $$t_{c,1}^{*}=\left\{\begin{array}{c} T-\frac{\ln\left(1+\frac{\mu_{r}-\mu_{w}}{b}\right)}{\mu_{r}-\mu_{w}},\\ \;\;\text{(single-party}\;\text{control},c\in \left\{q,w\right\})\\ T-\frac{\ln\left(1+C\frac{\mu_{r}-\mu_{w}}{b}\right)}{\mu_{r}-\mu_{w}},\\ \;(\text{mixed}\;\text{control,}\;c=\mathrm{mx}) \end{array}\right.$$

(see Supporting Information Sections 5 and 6 for derivation, especially see equations S109– S117, S119–S122, S133–S150, S140–S147). Under single‐party control, this switching time is equal for queen and worker control (i.e., $t_{q,1}^{*}=t_{w,1}^{*}$). Furthermore, in this case, it is identical to equation (6) of the clonal model of Macevicz and Oster (1976), by setting b=bR, $\mu_{w}=\mu$, and $\mu_{r}=\nu$ (see Supporting Information Section 13 for an overview of how our model relates to previous work).

It follows from equation (9) that the switch from the ergonomic to the reproductive phase under mixed control t mx ,1∗ depends on the potential for conflict C≥1. Furthermore, this switch happens earlier in the season under mixed control than under single‐party control (i.e., t mx ,1∗<tq,1∗=tw,1∗, see also Figure 1 for delayed dispersal and Figure 2 for direct dispersal, assuming queen monandry, i.e., C=1.5). The switching time t mx ,1∗ under mixed control happens earlier and, hence, the ergonomic phase is shorter if the potential for conflict *C* is larger. It turns out that the switching time t mx ,1∗ under mixed control is determined by the workers (see Supporting Information Section 8 for more detailed explanation). Equation (9) also implies that the onset of early reproduction under mixed control is more pronounced in poor habitats where resource acquisition rate is low and thus reproduction is slow (*b* is small), but colony per capita productivity still scales linearly as the colony grows (*b* is constant and does not depend on colony size). Increased mortality of workers (μ_w_) and decreased mortality of sexuals (μ_r_) also cause the time difference between optimal switching time and switching time under mixed control to be larger (see equation 9).

#### Production of males and queens in the reproductive phase

Under delayed dispersal, selection favors any allocation schedule that produces an allocation ratio of females and males at the end of the season, which is equal to the relatedness asymmetry. There are several uninvadable strategies that can satisfy this condition, the most simple one being the constant allocation, that is, proportional allocation to queens (during the reproductive phase) is given by

(10) $$a_{q}^{*}\left(t\right)=v_{f}^{*}\left(t\right)=\left\{\begin{array}{c} P_{q}=\frac{1}{2}\\ \;\text{(queen}\;\text{control}\;\text{and}\;\text{mixed}\;\text{control)},\\ P_{w}=\frac{2+M}{2(1+M)}\;\text{(worker}\;\text{control)}. \end{array}\right.$$

Under direct dispersal, selection favors the production of males before queens (protandry). This is because the reproductive success of males and queens depends asymmetrically on the time they are produced. The switching time $t_{c,2}^{*}$ from male production to queen production happens for M=1 when

(11) $$\frac{F_{c}\left(t_{c,2}^{*}\right)}{l_{q}\left(t_{c,2}^{*}\right)}=\left\{\begin{array}{c} R_{q}=1\\ \;\text{(queen}\;\text{control}\;\text{and}\;\text{mixed}\;\text{control)},\\ R_{w}=\frac{2+M}{M}\text{(worker}\;\text{control)}, \end{array}\right.$$

where the left‐hand side is the ratio of the cost to the benefit to (gene) fitness of producing a queen instead of a male at $t_{c,2}^{*}$ and the right‐hand side is the exchange rate between inseminated females and queens, which is given by the relatedness asymmetry (see Supporting Information Section 9 for proof). The cost of producing a queen instead of a male (at $t_{c,2}^{*}$) is equal to the potential mating success of a male (born at $t_{c,2}^{*}$), measured in the “currency” of expected number $F_{c}\left(t_{c,2}^{*}\right)$ of inseminated queens who survive until the end of the season. The benefit of producing a queen (at $t_{c,2}^{*}$) is equal to the probability $l_{q}\left(t_{c,2}^{*}\right)$ that she survives until the end of the season. Note that in a population where the queens mate *M* times, the expected number $MF_{c}\left(t_{c,2}^{*}\right)$ of surviving queens inseminated by males born at time $t_{c,2}^{*}$, has to be divided by the queen mating frequency *M* (because the focal male is expected to father only 1/M of the diploid offspring). Hence, equation (11) holds under any queen mating frequency *M*.

The queen is in control of the switch from male production to queen production under mixed control because under both queen and mixed control the switch happens at the time when producing a male instead of a surviving queen yields one surviving inseminated queen (recall equation 11). However, this does not imply that the switching time under queen control $t_{q,2}^{*}$ and mixed control t mx ,2∗ are equal and it follows from equation (11) that the switching time is

(12) $$t_{c,2}^{*}=\left\{\begin{array}{c} T-\frac{1}{\mu_{r}-\mu_{w}}\ln\left(\frac{b+\mu_{r}-\mu_{w}}{b+\left(1-P_{q}\right)\left(\mu_{r}-\mu_{w}\right)}\right)\\ \;\text{(queen}\;\text{control},c=q),\\ T-\frac{1}{\mu_{r}-\mu_{w}}\ln\left(\frac{b+\mu_{r}-\mu_{w}}{b+\left(1-P_{w}\right)\left(\mu_{r}-\mu_{w}\right)}\right)\\ \;(\text{worker}\;\text{control},c=w),\\ T-\frac{1}{\mu_{r}-\mu_{w}}\ln\left(2-\frac{b}{b+\frac{1}{2}C\left(\mu_{r}-\mu_{w}\right)}\right)\\ \;\text{(mixed}\;\text{control},c=\mathrm{mx}). \end{array}\right.$$

This shows that the switch to production of queens happens later under queen control than under worker control ($t_{q,2}^{*}>t_{w,2}^{*}$) because $P_{q}<P_{w}$ and it implies that more resources are expected to be allocated to queens under worker control than under queen control (since the length of the reproductive phase is the same under single‐party control, i.e., $t_{q,1}^{*}=t_{w,1}^{*}$). The switch to production of queens happens later under mixed control for higher values of the potential conflict *C*. Furthermore, the switch to queen production happens later when per worker productivity *b* is small, worker mortality rate μ_w_ is large, and the mortality rate μ_r_ of sexuals is large.

#### Switching times when the mortality rate of workers and sexuals is equal

In our model (1/b) can be loosely interpreted as the time it takes for one worker to help produce one offspring. We show in Supporting Information (see Sections 5.1.3, 5.1.4, and 6.2) that if the mortality rate of sexuals is roughly equal to the mortality rate of workers, then the switching time from the ergonomic to the reproductive phase $t_{c,1}^{*}$ under single‐party control (c={q,w}) approaches to the time (1/b) it takes for a worker to help produce an offspring before the season end (i.e., $t_{q,1}^{*}=t_{w,1}^{*}=T-1/b$); only the individuals produced at the end of the season are reproductive. However, under mixed control the switch happens *C* times earlier (i.e., t mx ,1∗=T−C/b). For example, when females mate only once (i.e., M=1 and C=1.5) then the switch to reproductive phase happens at time T−3/(2b).

### COLONY‐LEVEL TRAITS

#### Colony size at maturity and colony productivity

**Figure 5:**
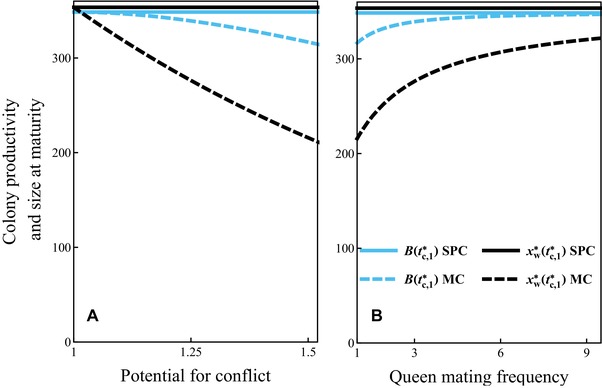
Colony productivity $B(t_{c,1}^{*})$ (blue lines) and size at maturity $x_{w}^{*}\left(t_{c,1}^{*}\right)$ (black lines) under single party (SPC, solid lines) and mixed control (MC, dashed lines) as a function of the potential for conflict *C* (panel a) and as a function of queen mating frequency *M* (panel b) for the uninvadable resource allocation schedule $u^{*}$. Recall that C=(2+M)/(1+M). Parameter values: b=0.07, $\mu_{w}=0.0015$, $\mu_{q}=\mu_{m}=0.001$, T=100.

During the ergonomic phase the number of workers in the colony grows exponentially until it reaches size $y_{w}^{*}\left(t_{c,1}^{*}\right)$ at maturity (i.e., maximum size, see Fig. 3 for delayed dispersal and Fig. 4 for direct dispersal). During the ensuing reproductive phase, sexuals are produced at rate $by_{w}^{*}\left(t\right)$. We define as colony productivity the total number $B\left(t_{c,1}^{*}\right)=y_{m}^{*}\left(T\right)+y_{q}^{*}\left(T\right)$ of (surviving) males and queens produced from $t_{c,1}^{*}$ until the end of the season. This can also be interpreted as the total sexual biomass produced in a colony (because we have assumed that males and females are equally costly to produce) and is a quantity often used as a fitness proxy in social insects (Wills et al. 2018). We show that under single‐party control the switching time $t_{c,1}^{*}$ from the ergonomic to the reproductive phase happens exactly at the time that maximizes colony productivity (see Supporting Information Section 7.2 for proof). Under mixed control the switch from the ergonomic to the reproductive phase happens earlier, especially for higher values of potential conflict *C*. Therefore, we predict that colony size at maturity and colony productivity would decrease with the increase in potential conflict *C* (that can be caused by, e.g., low queen mating frequency *M*). See also Figure 5 for illustration, Table 1 for the summary of parameter dependence, and Supporting Information Sections 7.1 and 7.2 for more technical details.

**Table 1:** Parameter dependence of colony resource allocation characteristics for biologically meaningful parameter values ($\mu_{w}>0$, $\mu_{r}>0$, $b>\mu_{w}$, and $b>\mu_{r}$)

We predict positive relationship between the allocation characteristics and the parameters listed under “Positive” column and negative dependence between the allocation characteristics and the parameters listed under “Negative” column. Here, “(MC)” and “(WC)” that follow after the parameter, emphasizes that these relations only hold for mixed or worker control, respectively.		
Parameter dependence of allocation characteristics		
Allocation characteristics	Positive	Negative
Switching times, $t_{c,1}^{*}$ and $t_{c,2}^{*}$	*M* (MC), *b*, μ_r_	*C* (MC), μ_w_
Colony size at maturity, $y_{w}^{*}\left(t_{c,1}^{*}\right)$	*M* (MC), *b*	*C* (MC), μ_w_
Colony productivity, $B(t_{c,1}^{*})$	*M* (MC), *b*, μ_r_	*C* (MC), μ_w_
Sex‐allocation ratio for delayed dispersal, $S_{c}$ (proportional allocation to queens)	*C* (WC)	*M* (WC)
Sex‐allocation ratio for direct dispersal, $S_{c}$ (proportional allocation to queens)	*C* (WC), *M* (MC)	*M* (WC), *C* (MC), μ_r_, μ_w_, *b*

#### Sex ‐allocation ratio

We define the overall sex‐allocation ratio $S_{c}$ at the evolutionary equilibrium as the proportion of the colony resources allocated to queens from the resources allocated to sexuals over the entire season (irrespective of whether they survive to reproduce), where the subscript c∈{q,w, mx } emphasizes the dependence on the scenario of genetic control (see Supporting Information Section 7.3 for a formal definition). $S_{c}$ can be interpreted as the overall proportion of queens among sexuals produced in the colony because we assume that males and queens are equally costly to produce.

Under delayed dispersal, the overall sex‐allocation ratio is given by (Supporting Information Section 7.3, equations S172– S175)

(13) $$S_{c}=\left\{\begin{array}{c} P_{q}\;\text{(queen}\;\text{control}\;\text{and}\;\text{mixed}\;\text{control)},\\ P_{w}\;\text{(worker}\;\text{control)}. \end{array}\right.$$

Hence, under delayed dispersal the overall sex‐allocation ratio is given by the proportional relatedness asymmetry (via equation 10 and recall equation 7). It follows from equation (13) that the prediction for the uninvadable overall sex‐allocation ratio under single‐party control is equal to the corresponding prediction from the standard static models of sex‐allocation theory (Trivers and Hare 1976; Boomsma and Grafen 1991; Reuter and Keller^2001^).

Under direct dispersal, the overall sex‐allocation ratio is given by (Supporting Information Section 7.3, equations S176– S178)

(14) $$S_{c}=\frac{e^{-\mu_{w}t_{c,2}^{*}}-e^{-\mu_{w}T}}{e^{-\mu_{w}t_{c,1}^{*}}-e^{-\mu_{w}T}}.$$

Note that the overall sex‐allocation ratio under direct dispersal, in contrast to delayed dispersal, depends also on other life‐history characteristics of the species and not only on the proportional relatedness asymmetry in the colony (which enters into the equation via $t_{c,1}^{*}$ and $t_{c,2}^{*}$).

**Figure 6:**
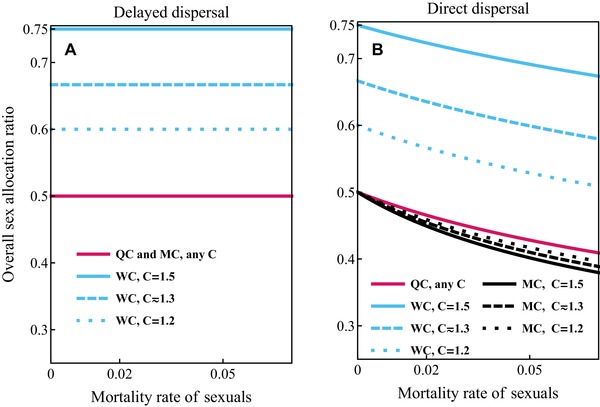
Overall proportional sex‐allocation ratio $S_{c}$ (proportional investment into queens) as a function of mortality rate of the sexuals μ_r_ for different values of potential for conflict *C*. Panel (a): delayed dispersal; queen and mixed control (QC and MC, red lines), worker control (WC, blue lines). Panel (b): direct dispersal; queen control (QC, red lines), worker control (WC, blue lines), mixed control (MC, black lines). Other parameter values: b=0.07, $\mu_{w}=0.015$, T=100. Note that classical results from static models (e.g., Reuter and Keller 2001) only coincide with these results under delayed dispersal and single‐party control.

**Figure 7:**
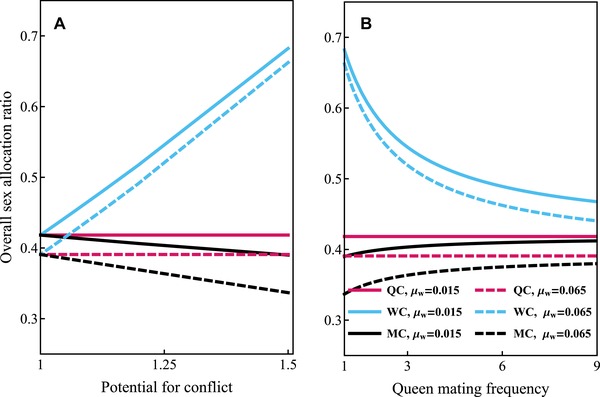
Overall proportional sex‐allocation ratio $S_{c}$ (proportional investment into queens) under direct dispersal as a function of the potential for conflict *C* (panel a) and queen mating frequency *M* (panel b) for different values of mortality of workers μ_w_. Queen control (QC, red lines); worker control (WC, blue lines); mixed control (MC, black lines). Parameter values: b=0.07, $\mu_{r}=0.06$, T=100.

The overall sex‐allocation ratio is more male‐biased under direct dispersal than under delayed dispersal and compared to results from static models of sex‐allocation theory (e.g., Trivers and Hare 1976; Boomsma and Grafen 1991). Furthermore, the male‐bias is more pronounced under mixed control than under single‐party control. We illustrate in Figures 6 and 7 that this male bias can be substantial for higher values of mortality rates of sexuals and workers, for example, S mx ≈0.35 for mixed control under monandry, compared to S mx =0.5 under delayed dispersal and S mx ≈0.56 according to the corresponding static allocation model (see Table S3 in Supporting Information Section 12, see also Table 1 for a summary of how $S_{c}$ depends qualitatively on the parameters of the model). Mortality of sexuals increases male‐biased allocation because it increases the mating success of males produced before the emergence of queens (see Discussion for more elaborate explanation).

This effect of mortality in inducing male‐biased allocation is stronger under mixed control, especially for higher values of the potential for conflict *C*, because proportionally more sexuals die when the reproductive phase is longer (as it is under mixed control for high values of *C*). Hence, under mixed control and direct dispersal, the overall proportional allocation to queens is lower for higher values for the potential for conflict *C* (i.e., for lower values of queen mating frequency *M*, see Figs. 6 and 7).

Regardless of the order in which sexuals are produced, the primary sex‐allocation ratio $u_{f}^{*}\left(t\right)$ during the reproductive phase determines the overall sex‐allocation ratio. Hence, the queen is in control of the overall sex‐allocation ratio under mixed control (see also Supporting Information Section 8 for more detailed explanation).

### UNEQUAL MORTALITY RATES OF SEXUALS

We now discuss how relaxing the assumption of equal mortality ($\mu_{q}=\mu_{m}=\mu_{r}$) used in the derivation of the above results qualitatively affects these results. From further analysis (Supporting Information Section 5.2) and our numerical solutions, we find that under delayed dispersal, if the mortality rate of queens and males is not equal, then the sex with the lower mortality rate should be produced earlier, such that by the end of the season the sex ratio of queens to males would be given by $R_{c}$ under single party control and *R*_q_ under mixed control (assuming that males and queens are equally costly to produce).

We also find that the main conclusions of our results under direct dispersal hold qualitatively if $R_{c}\mu_{q}\geq \mu_{m}$ under single‐party control and $R_{q}\mu_{q}\geq \mu_{m}$ under mixed control. Under direct dispersal, if $R_{c}\mu_{q}<\mu_{m}$ then the overall sex‐allocation under single‐party control can be more female‐biased than the static models of sex‐allocation theory predict (e.g., Trivers and Hare 1976; Boomsma and Grafen 1991). Similarly, if $R_{q}\mu_{q}<\mu_{m}$ then the overall sex‐allocation under mixed control and direct dispersal can be female‐biased. Furthermore, we find that under mixed control, if the mortality of queens is significantly lower than that of males, then males and queens are produced simultaneously after the switch to the reproductive phase, until there is a switch to producing only females (see Supporting Information Section 6.3).

## Discussion

Ontogenetic development of social insect colonies causes behavioral trait expressions of individuals to be necessarily time‐dependent (Oster and Wilson 1979). In this article, we formulated a mathematical model to analyze how sex‐allocation conflict affects the dynamic (time‐dependent) allocation of resources to workers, queens, and males in annual eusocial monogynous species. We have considered three alternative scenarios of control of colony trait expression (full queen, full worker, and mixed control) and two alternative scenarios of dispersal of sexuals: direct dispersal after eclosion (common among bees and wasps) and delayed dispersal at the end of the season, which resembles the life history of species, where nuptial flights are synchronized (more commonly found in ants, e.g., see Heinze 2016, and references therein). Our model extends static allocation models with genetic conflict and dynamic allocation models without conflict and it allows to shed light on a number of questions about colony ontogeny, such as: how does sex‐allocation conflict affect colony growth? How does sexual selection affect the production of sexuals? Which party wins the sex‐allocation conflict?

Our results suggest that the marginal benefit of allocating a unit resource to a queen rather than to a male is weighed by the relatedness asymmetry, regardless of any details of colony life‐cycle or growth dynamics, thereby generalizing the standard static first‐order condition of sex‐allocation theory (e.g., Boomsma and Grafen 1991; Taylor and Frank 1996) to any pattern of colony ontogeny. Solving the first‐order condition under our specific life‐cycle assumptions using optimal control theory (a nontrivial task, see Supporting Information Sections 5 and 6), we find that selection tends to favor a colony resource allocation schedule that consists of two qualitative phases. First, an ergonomic phase with production of only workers, which determines the colony size at maturity. Second, a reproductive phase with resource allocation to queens and males, which determines the colony productivity and overall sex‐allocation ratio. Sexuals can be produced according to various schedules, possibly including switching between producing only males or females (or vice versa), depending on life‐cycle assumptions. Colony traits such as the switching times between different phases of colony growth, maximum colony size, colony productivity, and overall sex‐allocation ratio are influenced by the assumptions about the genetic control of resource allocation traits and individual dispersal behavior.

### HOW DOES SEX‐ALLOCATION CONFLICT AFFECT COLONY GROWTH?

Our results confirm earlier predictions derived from dynamic resource allocation models (Macevicz and Oster 1976; Ohtsuki and Tsuji 2009) that colony resource allocation should consist of an ergonomic phase and a reproductive phrase. During the ergonomic phase, the marginal return of workers is higher than the return of investment into sexuals. Workers have a higher early marginal return because colony productivity rate ($by_{w}$) increases linearly with colony size (hence exponentially during the ergonomic phase), allowing for the production of more sexuals later in the season. Sexuals have a lower early marginal return because they need to survive (queens need to survive until the end of the season and males need to survive until they can reproduce with the surviving queens). The colony switches from the ergonomic to the reproductive phase when producing workers no longer yields the highest marginal return.

We find that under mixed control, colonies switch earlier to the reproductive phase than under single‐party control. This early switch evolves because under mixed control the queen controls the sex‐allocation ratio (for why this is so, see section “Which party wins the sex allocation conflict?” below), meaning that workers cannot increase allocation to queens during the reproductive phase, even though producing more queens would increase the fitness of genes residing in workers. Hence, workers start rearing female eggs (destined to become workers under single‐party control) into queens earlier, to increase the allocation to queens. Hence, asymmetric control over the sex‐allocation ratio causes the switching time to the reproductive phase to be controlled by the workers (see also Supporting Information Section 8 for more technical explanation).

Colony size at maturity and colony productivity are expected to be smaller under mixed control than under single party control. Under single‐party control the colony productivity is maximized, but not under mixed control (see Supporting Information Section 7.2 for proof and Fig. 5). This is so because in the latter case the switch to the reproductive phase occurs earlier, causing colony size at maturity to be smaller (there is less time for worker numbers to increase exponentially during the ergonomic phase). Therefore, there are fewer workers to produce sexuals in the reproductive phase, which results with a decline in colony productivity (colony‐level cost of sex‐allocation conflict).

A loss in colony productivity due to sex‐allocation conflict was already predicted using a static (Reuter and Keller 2001) and a dynamic allocation model assuming delayed dispersal (Bulmer 1981). But for the latter model, the outcome of the resource allocation conflict is different from ours. Indeed, Bulmer (1981) concluded that colonies die one generation before the end of the season if the sex allocation at the population level is biased toward queens because the queens are producing only males in the penultimate generation. His conclusion relied on the assumption that colony growth is divided into discrete generations, such that worker generations within a season do not overlap and in his model he only considered two generations before the end of the season. Our analysis not only extends the results of Bulmer (1981) to less restrictive life‐cycle assumptions and to direct dispersal of sexuals, but it also provides quantitative predictions for the switching time from the ergonomic to the reproductive phase. Indeed, we predict that the premature switch from the ergonomic to the reproductive phase is earlier in species where the resource acquisition rate is low, the mortality rate of workers is high and that of sexuals low. We also show that the switching times from the ergonomic to the reproductive phase under mixed control are equal for both delayed dispersal and direct dispersal. This implies that sexual selection and the evolution of protandry do not have an effect on the cost of sex‐allocation conflict that manifests itself through loss of colony productivity.

The switching time to the reproductive phase under mixed control depends on the potential for conflict *C*, which is the ratio of party‐specific proportional contribution of genes through queens to the gene pool in the distant future (equation 8), and a decreasing function of the mating number *M* of a queen. Our results imply that colonies with lower potential for conflict *C* are expected to grow larger and have higher colony productivity. Similar effects can be expected to hold for other factors that reduce the queen‐worker conflict over sex allocation, for example, polygyny of related queens or worker production of male eggs (Reuter and Keller 2001; Ratnieks et al. 2006). We have assumed monogyny, but allowing for multiple queens per colony should be a relatively straightforward extension to our model. Our analysis implies that polyandry is expected to evolve under mixed control, given that the workers are able to assess the mating frequency of the queen (Pamilo 1991b). However, empirical evidence suggests that polyandry is generally less common in annual eusocial insects but has been found, for example, in *Polistes* (Seppä et al. 2011) and *Vespula* (Johnson et al. 2009).

The so‐called “bang‐bang” schedule of colony growth, such that allocation to workers and sexuals never occurs simultaneously, represents a general life‐history principle of growth and reproduction in annual organisms for which productivity rate scales linearly with size and environmental fluctuations that can cause variations in the length of the season or food availability are small (Cohen 1971; King and Roughgarden 1982). A key result of our analysis is that the sex‐allocation conflict does not affect the overall shape of the colony growth curve, but only the time of the switch between growth and reproduction. This is not an obvious result because trade‐offs between producing different types of individuals are not linear. It has been shown before (assuming clonal reproduction) that selection favors a singular control (sometimes called a graded control; i.e., workers and sexuals are produced simultaneously) if the productivity rate (i.e., $by_{w}$) scales nonlinearly with colony size, such that $b\equiv b(y_{w})$ (Beekman et al. 1998; Poitrineau et al. 2009), but not for environmental fluctuations acting alone (Mitesser et al. 2007b). The properties of the relationship between productivity rate and colony size affect the way the marginal value of producing a worker changes over time, but not the marginal value of producing queens and males. In principle, this could affect the outcome of the sex‐allocation conflict and it would be interesting to see if the results of our model change when the productivity rate would scale nonlinearly with colony size.

Inherently, our model assumes that individuals in the colony possess some physiological mechanism that enables them to estimate the timing of the switch from the ergonomic phase to the reproductive phase. Currently, the underlying mechanism behind the timing of the switch from the ergonomic to the reproductive phase is not known (but it has been shown that *Bombus terrestris* queens are able to control the switching time endogenously, Holland et al. 2013). Nevertheless, the framework of our model can be used to also study the evolution of eusociality, when we allow for the brood to have control over their own developmental fate. Current models that study the emergence of eusociality that explicitly track colony growth usually fix the switch from ergonomic to reproductive phase to happen at arbitrary size of the colony (e.g., Avila and Fromhage 2015). Hence, extending our model to study evolution of eusociality could explain how life‐history interacts with other mechanisms that are known to drive the evolution of eusociality.

### HOW DOES SEXUAL SELECTION AFFECT THE PRODUCTION OF SEXUALS?

Our model predicts simultaneous production of queens and males under delayed dispersal and protandry (males produced before females) under direct dispersal. Under delayed dispersal, both males and queens have to survive until the end of the season to mate and their reproductive success depends symmetrically on the time that they are produced. Under direct dispersal, males have to survive until there are females available to mate, whereas queens have to survive until the end of the season. This asymmetry leads to protandry.

Our prediction about the evolution of protandry relies on the assumption that the females mate immediately and indiscriminately after dispersal with the males currently in the mating pool. However, there is some evidence of female choice in some social insects (Baer 2003, and references therein). Nevertheless, there is also evidence that earlier emergence of males can give them an advantage in mating success through precopulatory sexual behaviors or through the use of mating plugs (Foster 1992; Baer et al. 2000; Baer 2003, 2014).

### WHICH PARTY WINS THE SEX‐ALLOCATION CONFLICT?

We show that the queen wins (more accurately, the genes in queens win) the sex‐allocation conflict because the evolution of distinct phases of colony growth constrains the ability of workers to manipulate the overall sex‐allocation ratio. Indeed, during the reproductive phase, the ratio at which the queen lays the female versus male eggs determines the overall sex‐allocation ratio because workers can only influence the developmental fate of the female eggs. Therefore, the only option for workers to increase the allocation to queens is to switch to the reproductive phase earlier at the expense of reduced colony productivity, whereas queens, regardless of the early switch, can always further affect the sex ratio without disturbing colony productivity.

The evolution of different phases of colony growth is thus crucial as it decouples the trade‐offs experienced by the queens. During the ergonomic phase, there is a latent trade‐off between producing males versus workers (because workers rear all the female eggs into workers), whereas during the reproductive phase there is a trade‐off between producing queens versus males (because workers rear all the female eggs into queens). The distinct phases of colony growth also decouple how queens and workers can affect the allocation decisions in the colony, impeding the ability of workers to influence the overall sex allocation during the reproductive phase and the ability of queens to influence the proportional allocation to workers versus sexuals (see also Supporting Information Section 8 for more detailed explanation). Our results thus suggest that the queen is always expected to win the sex‐allocation conflict, as long as workers and sexuals are produced during separate phases of colony growth and workers can only influence the developmental fate of the female eggs.

### THE OVERALL SEX‐ALLOCATION RATIO

In our model, the overall sex‐allocation ratio depends on the scenario of dispersal of sexuals. Under mixed control, the overall sex‐allocation ratio is expected to be even under delayed dispersal and male‐biased under direct dispersal (given that the mortality rate of males and queens is equal). Under single‐party control and delayed dispersal, the overall sex‐allocation ratios predicted by our model are in accordance with the classical static models (e.g., Trivers and Hare 1976; Boomsma and Grafen 1991) and do not depend on the life‐history characteristics of the species (e.g., mortality rate of sexuals or workers). However, under direct dispersal, we observe more male‐biased overall sex‐allocation ratios than occur in the static models of sex‐allocation theory (e.g., Trivers and Hare 1976; Boomsma and Grafen 1991), especially for higher mortality rates of sexuals (see Fig. 6) and lower mortality rates of workers (see Fig. 7).

More male‐biased sex‐allocation ratios evolve under direct dispersal because mortality affects the co‐evolution of protandry (that evolves due to sexual selection on males) and sex‐allocation ratio. The sex‐allocation ratio is determined by the switching time from male production to queen production. This happens when producing a male yields $R_{c}$ (surviving) inseminated queens, instead of producing a (surviving) queen. Hence, the relative mating success of males compared to the survival probability of queens determines the switching time from male production to queen production. When mortality of sexuals is high, males produced later in the season (just before the emergence of queens) have higher mating success because there are fewer surviving males to compete with. Hence, higher mortality of sexuals delays the switch to queen production because it increases the mating success of males (see Supporting Information Section 9 for a more detailed analysis and explanation). Our result that mortality affects the sex‐allocation ratio appears to be at variance with Fisher's (1930) result that mortality after parental investment (either differential between the sexes or not) should not affect the uninvadable sex‐allocation ratio (see, e.g., West 2009, pp. 19–20). The reason for this apparent discrepancy is that, in our model, mortality causes resources that are invested into sexuals earlier to yield lower fitness returns (because early‐produced sexuals have a lower chance to contribute to the next generation). So, mortality causes males to be produced more cheaply (at a time when allocating resources yield smaller returns). Hence overproduction of males under higher mortality is in fact consistent with Fisher's prediction that more offspring should be produced of the cheaper sex.

Under direct dispersal, the overall sex‐allocation ratio is more male‐biased for mixed control than for queen control, even though for both queen and worker control, the switch from male production to queen production happens when producing a male instead of a surviving queen yields one surviving inseminated queen. This is because, for mixed control, the reproductive phase is longer during which proportionally more males die before they can mate, which increases the mating success of males produced later. This is why the overall allocation is more male‐biased under mixed control for higher values of mortality of sexuals (see Fig. 6) and for other life‐history characteristics that cause the reproductive phase to be longer, such as higher values of the mortality rate of workers μ_w_ (see Fig. 7). Hence, we find that in protandrous species, proportionally more resources are expected to be allocated into producing males.

Surprisingly, under direct dispersal and mixed control the overall sex‐allocation ratio *S*_mx_ becomes more male‐biased as the workers become more related to the female brood (their sisters) (i.e., if the potential for conflict *C* increases or the queen mating frequency *M* decreases, see Fig. 7). This prediction follows from the combined effect of protandry under direct dispersal and a longer duration of the reproductive phase for higher values of the potential for conflict under mixed control. If workers are more related to the female brood (e.g., for higher values of the potential conflict *C*), then the mating success of males produced later is higher because proportionally more males have died due to early switch to the reproductive phase. For these reasons, worker relatedness to female brood is expected to correlate negatively with the proportional investment into queens when resource allocation is under mixed control. This prediction contradicts standard results from the static models of sex‐allocation theory (Trivers and Hare 1976; Boomsma and Grafen 1991) that predict the opposite correlation. We expect that other factors that reduce the queen‐worker conflict over sex‐allocation have qualitatively similar effects on overall proportional allocation to queens.

Most comparative studies about population‐wide sex allocation of eusocial Hymenoptera come from ants, where sex allocation is mostly female‐biased (Bourke and Franks 1995; Sundström et al. 1996; Ratnieks et al. 2006), although it is not universal (Helms 1999; Helms et al. 2000; Passera et al. 2001; Fjerdingstad et al. 2002). However, most ant species are perennial and their life cycles diverge in many respects from the assumptions of our model. In bumble bees, who are annual and mostly monogynous species, the population‐wide sex allocation tends to be overwhelmingly male‐biased (Bourke 1997). Indeed, Bourke (1997) found that the median proportional allocation to queens is only 0.32 (range 0.07–0.64) among 11 populations of seven bumble bee species. Interestingly, Johnson et al. (2009) found that in a social wasp (*V. maculifrons*) nestmate relatedness is negatively associated with overall investment into queens, which would be in accordance with our model for mixed control under direct dispersal with male protandry (see Fig. 6). However, these results arise from a dataset where the queens have a relatively high mating frequency and the variation between mating frequencies is not very large (hence, the effect size is not very large) and male protandry in that species is not entirely clear (Johnson et al. 2009).

### STATIC AND DYNAMIC APPROACHES TO RESOURCE ALLOCATION CONFLICTS

Corresponding static and dynamic models can make different predictions for the outcome of the conflict. This can be seen when comparing the predictions of our model under delayed dispersal with the predictions of a corresponding static model by Reuter and Keller (2001). See Supporting Information Section 12, for a proof that our model is indeed comparable to that of Reuter and Keller (2001), even though there is a slight deviation in the assumption about how productivity scales with colony size (because this assumption does not affect qualitatively their results). We followed their approach on modeling conflict by way of using mixed control of colony allocation traits, but our result that the queen wins the sex‐allocation conflict contradicts with theirs. Indeed, they predicted that the sex‐allocation ratio under mixed control is intermediate between sex‐allocation ratios predicted for queen and worker control (the exact values depending on the assumption about how productivity scales with colony size). This contradiction arises because in our dynamic model the sex‐allocation ratio is determined during the reproductive phase by the queen, while in the model of Reuter and Keller (2001) behavioral decisions cannot vary over time, meaning that the two parties make their decisions simultaneously for the whole season *T*. Hence, this way of modeling links all the allocation decisions together to happen simultaneously, which leads to the result that workers can influence the sex‐allocation ratio by rearing some worker–destined female brood into queens.

It has been shown by Pen and Taylor (2005) that if the two parties make their allocation decisions sequentially (the so‐called Stackelberg equilibrium, such that the queen acts first and workers respond), then the queen is expected to win the sex‐allocation conflict even assuming static resource allocation decisions. Pen and Taylor (2005) studied a static resource allocation model similar to the model of Reuter and Keller 2001), but they also looked at the effect of information exchange between the two parties. Although they arrived at a conclusion similar to ours about the overall sex‐allocation ratio, our result implies that the workers do not have to have the information about the ratio at which the queen lays the male to female eggs.

Reuter and Keller (2001) also generally argue that complete control by a single party is not evolutionarily stable because the conflict over sex‐allocation strongly selects for the other party to manipulate the sex allocation, leading to a stable evolutionary equilibrium where the sex allocation is intermediate between the predicted evolutionary outcomes for full control of the two parties. However, under the dynamic model, we show that under the assumptions of mixed control, an intermediate sex allocation will not evolve.

### CONCLUSION

We showed that when dynamic properties of resource allocation are considered, sex‐allocation conflict can substantially affect colony ontogeny, and thus the overall patterns of growth and productivity. Helanterä (2016) has argued that life‐history trade‐offs may be easier traits to conceptualize as organismal traits (i.e., traits evolving like group‐selected adaptations), as opposed to traits more heavily contingent on conflicts among genes in different individuals, such as traits involving sex allocation and dispersal behavior. In contrast, our model suggests that colony life‐history traits can generally not be viewed in isolation from traits that are influenced by genetic conflicts, and hence both “morphology” and “physiology” of a colony are likely to be affected by them, leading to a general breakdown of the “organismic” perspective of eusocial insect colonies.

Associate Editor: E. Kisdi

Handling Editor: P. Tiffin

## Supporting information


**Figure S1**. Uninvadable proportional allocation (under delayed dispersal) to workers $a_{w}^{*}\left(t\right)=u_{f}^{*}\left(t\right)\left(1-u_{q}^{*}\left(t\right)\right)$ (black asterisks), queens $a_{q}^{*}\left(t\right)=u_{f}^{*}\left(t\right)u_{q}^{*}\left(t\right)$ (red circles), and males $a_{m}^{*}\left(t\right)=\left(1-u_{f}^{*}\left(t\right)\right)$ (blue circles).
**Figure S2**. Number of individuals produced in a colony following the uninvadable resource allocation schedule u* under delayed dispersal.
**Figure S3**. Uninvadable proportional allocation (under direct dispersal) to workers $a_{w}^{*}\left(t\right)=u_{f}^{*}\left(t\right)\left(1-u_{q}^{*}\left(t\right)\right)$ (black), queens $a_{q}^{*}\left(t\right)=u_{f}^{*}\left(t\right)u_{q}^{*}\left(t\right)$ (red), and males $a_{m}^{*}\left(t\right)=\left(1-u_{f}^{*}\left(t\right)\right)$ (blue).
**Figure S4**. Number of individuals produced in a colony following the uninvadable resource allocation schedule u* under direct dispersal.
**Table S1**. Candidate optimal controls and conditions for the signs of switching functions for all possible regimes of colony growth.
**Table S2**. Uninvadable allocation into queen, males, and workers and the overall sex‐allocation ratio $S_{c}$ (proportional allocation to queens from resources allocated to sexuals) predicted by Reuter and Keller (2001).
**Table S3**. Uninvadable allocation into queen, males, and workers and the overall sex‐allocation ratio $S_{c}$ (proportional allocation to queens from resources allocated to sexuals) predicted by a static model similar to Reuter and Keller (2001), assuming that colony productivity scales linearly with colony size.

## Data Availability

There is no data to be archived.
